# *In-vitro* safety and off-target profile of the anti-parasitic arylmethylaminosteroid 1o

**DOI:** 10.1038/s41598-020-64382-w

**Published:** 2020-05-05

**Authors:** Leonard Blum, Sheraz Gul, Thomas Ulshöfer, Marina Henke, Reimar Krieg, Isabell Berneburg, Dominique Thomas, Sandra Trautmann, Jennifer Kurz, Joachim Geyer, Gerd Geisslinger, Katja Becker, Michael J. Parnham, Susanne Schiffmann

**Affiliations:** 1 Fraunhofer Institute for Molecular Biology and Applied Ecology IME, Branch for Translational Medicine and Pharmacology (TMP), Theodor-Stern-Kai 7, 60596 Frankfurt/Main, Germany; 2 pharmazentrum frankfurt/ZAFES, Department of Clinical Pharmacology, Goethe-University Hospital Frankfurt, Theodor-Stern-Kai 7, 60590 Frankfurt/Main, Germany; 3 Biochemistry and Molecular Biology, Interdisciplinary Research Center, Justus-Liebig-University, Heinrich-Buff-Ring 26-32, 35392 Giessen, Germany; 4 Fraunhofer Institute for Molecular Biology and Applied Ecology IME – ScreeningPort, Schnackenburgallee 114, 22525 Hamburg, Germany; 5 Department of Anatomy II, University Hospital Jena, Teichgraben 7, 07743 Jena, Germany; 6 Faculty of Veterinary Medicine, Institute of Pharmacology and Toxicology, Justus-Liebig-University, Schubertstraße 81, 35392 Giessen, Germany

**Keywords:** Target validation, Toxicology, Drug safety

## Abstract

Parasite-mediated diseases like malaria and schistosomiasis are growing health problems worldwide and novel drug candidates are urgently needed. In this study, the *in-vitro* safety profile of steroid compound 1o (sc1o), effective against the parasites *Plasmodium falciparum* and *Schistosoma mansoni* with an IC_50_ value of 5 nM, was characterized. We assessed viability/proliferation, apoptosis and cell cycle tests to determine the cytotoxic profile of sc1o in cancer cells. The mutagenic potential was determined with the AMES test. To identify off-target effects we investigated whether sc1o interacts with safety-relevant molecules such as cytochrome P450 (CYP) enzymes, phosphodiesterases (PDE), histone deacteylases (HDAC) and human ether-a-go-go related gene (*h*ERG). Furthermore, to predict the potential bioavailability of sc1o, its effect on Caco-2 cell barrier integrity, by measurement of the transepithelial electrical resistance (TEER), was determined. Sc1o at 25 µM reduced cell viability, probably through cell-cycle arrest, but did not induce apoptosis in cancer cells. No adverse off-target effects nor mutagenic potential of sc1o were observed. Furthermore, sc1o did not disturb the integrity of the cell barrier, but exhibited low membrane permeability, apparently due to cell adherence. In conclusion, sc1o up to 10 µM showed a good *in-vitro* safety profile.

## Introduction

Parasite mediated diseases such as malaria (*Plasmodium)* and schistosomiasis (*Schistosoma)* are growing global health challenges. The WHO reported 228 million cases of malaria worldwide in 2018 (WHO 2018), while schistosomiasis affects approximately 200–250 million people, mostly in developing countries^1–3^. Since no effective malaria vaccine is available, chemotherapy remains an important weapon against malaria. Standard treatment is commonly based on artemisinin combination therapies, but reports of artemisinin-resistant parasites stress the urgent need for new therapeutic approaches^4,5^. A common therapy approach to schistosomiasis is the use of the anthelminthic drug praziquantel^6^. Frequent use of praziquantel increases the risk of development of resistance mechanisms^7,8^. Therefore, new therapeutic approaches to infections with both parasites are urgently needed.

The steroid compound 1o (sc1o) is a new lead compound with promising activity against intraerythrocytic stages of chloroquine-sensitive and resistant *Plasmodium falciparum* parasites (IC_50_ 1–5 nM)^9^. Furthermore, in *P. berghei* infected mice, oral administration of sc1o drastically reduces parasitaemia and seems to cure the animals^9^. Sc1o shows also remarkable activity against the blood-feeding trematode parasite *Schistosoma mansoni*^9^. With such good activity against the pathogen, an adequate safety and pharmacokinetic profile is crucial for further potential development as a drug candidate. The *in-vitro* safety profile should include, among others, viability, apoptosis, mitochondrial activity, off-target effects and the AMES test^10^. For the pharmacokinetic profile, the Caco-2 cell barrier assay *in-vitro* can be used to gain initial insights into the expected bioavailability of the drug^11^. Many drugs are metabolized by cytochrome P450 (CYP) enzymes. This class has more than 50 enzymes, however, six of them metabolize 90 percent of drugs, with the three most significant enzymes being CYP3A4, CYP1A2 and CYP2D6^12^. Therefore, we tested whether sc1o interacts with these CYP proteins. To obtain a broad off-target profile of sc1o, further safety-relevant proteins such as the cAMP specific phosphodiesterases (PDE) (PDE4, PDE7, PDE8), histone deacteylases (HDAC) and human ether-a-go-go related gene (*h*ERG)^13^ were selected. In this current study, these aspects were investigated in cell culture systems to assess whether sc1o is a suitable drug candidate for further development.

## Results

### Sc1o at high concentrations reduced cell viability in colon cancer cells

**Figure 1:**
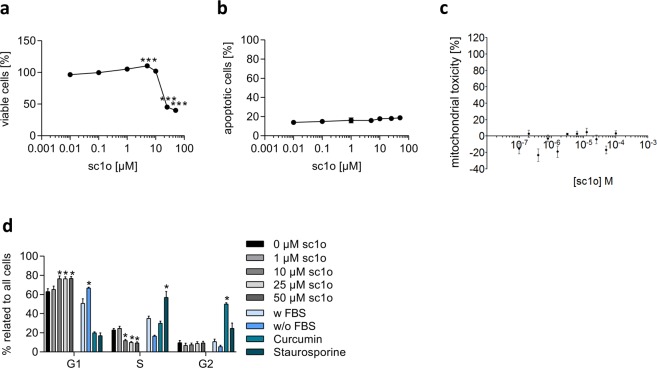
Impact of sc1o on cell viability, mitochondrial toxicity and cell cycle. (**a**) For the cell viability assay, HCT 116 cells were incubated with 0.01–50 µM of sc1o over 24 h. To calculate cell viability, the absorbance of DMSO-treated cells was set to 100%, and the sc1o samples were correlated to the absorbance of DMSO value. (**b**) For the apoptosis assay, HCT 116 cells were incubated with 0.01–50 µM of sc1o over 24 h. To calculate the apoptotic rate, the number of apoptotic cells were correlated to the total number of cells. (**c**) Dose response curves for mitochondrial toxicity by sc1o. The MitoTracker Red CMXRos dye was used to stain mitochondria in live cells. Analysis of mitochondrial toxicity was performed with the Opera imaging system. Sc1o was profiled in the assay in 11-point concentration–response format with the raw data normalized using the positive control (1 µM valinomycin) and negative control (DMSO). (**d**) For cell cycle analysis, HCT 116 cells were incubated with sc1o in concentration as indicated or with controls over 24 h. Using flow cytometry, the amount of cells in the various cell cycle phases (G1, S, G2) was determined. The flow cytometry data was analysed with a specific cell cycle analysis method from FlowJo software. For statistical analysis, one-way (**a, b**) or two-way (**d**) ANOVA with Dunnett’s comparison tests were used. The experiments were performed in triplicate and repeated three times. *p < 0.05, ***p < 0.001 indicate significant differences between sc1o and DMSO data.

A prerequisite for the preclinical characterization of a drug is cytotoxicity testing, which can include proliferation, apoptosis, and cell cycle assays. We investigated whether sc1o influences cell viability and proliferation. Hughes *et al*. recommend for ‘no toxicity’ concentrations, at least 50-fold higher values than the IC_50_ of the test drug^10^. Since sc1o had an IC_50_ value of about 5 nM on *P. falciparum*^9^, we used a range from 0.01–50 µM. Cell viability/proliferation was assessed with the WST-1 assay in the colon cancer cell line HCT 116. At concentrations up to 10 µM, sc1o did not reduce cell viability of HCT 116 cells. However, at 25 µM and 50 µM, sc1o reduced cell viability to about 35% and 40%, respectively (Fig. 1a).

### Sc1o did not induce apoptosis or mitochondrial toxicity

We next investigated whether the reduced cell viability mediated by sc1o was due to induction of apoptosis. Caspase 3 activation is a crucial component of the apoptotic machinery^14^. Therefore, apoptosis was detected by activated caspase 3 and staining nuclei (Draq5). HCT 116 cells were incubated with sc1o in a range of 0.01–50 µM or with DMSO for 24 h. Sc1o and DMSO induced comparable apoptotic rates, indicating that sc1o itself did not induce apoptosis in HCT 116 cells (Fig. 1b). Furthermore, we checked whether sc1o induces mitochondrial toxicity. Renal carcinoma cells were treated with various concentrations of sc1o, and the mitochondrial toxicity was detected using MitoTracker probes, which accumulate only in active mitochondria. However, sc1o did not influence the activity of mitochondria (Fig. 1c).

### Sc1o at high concentrations induced a G1-block

Another mechanism for reduced cell viability caused by sc1o could be the interaction of sc1o with the cell cycle. Cell cycle regulation was measured by DNA-staining the cells with propidium iodide and detecting the DNA content and size of the cells via flow cytometry. To analyse the data, the percentages of cells in the different phases (G1, S, and G2) were calculated. As a positive control, G1, S, and G2 blocks were induced with starvation (medium without fetal calf serum (FCS)), 0.2 µM of staurosporine, and 20 µM of curcumin, respectively (Fig. 1d). Interestingly, 10 µM sc1o and higher concentrations also led to a significant increase in cells in the G1 phase and to a significant decrease in cells in the S phase (Fig. 1d). These data indicate that sc1o induced a G1 block at higher concentrations (>10 µM), a mechanism possibly responsible for the observed reduction in cell viability at these concentrations.

### Sc1o did not inhibit off-target enzymes

**Figure 2:**
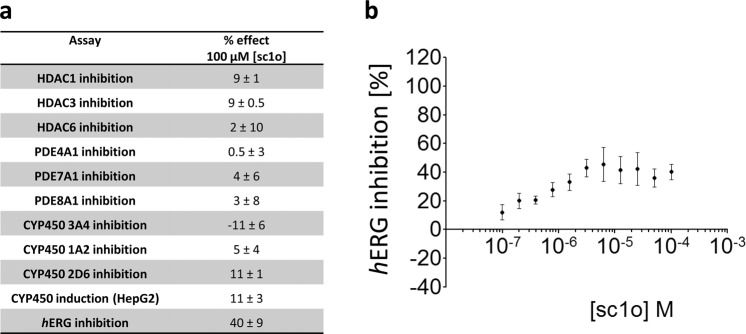
Off-targets effects of sc1o. (**a**) For the safety profile and off-target studies of sc1o (CYP3A4, CYP1A2, CYP2D6 inhibition, CYP induction, *h*ERG, HDAC1, HDAC3, HDAC6, PDE4A1, PDE7A1, and PDE8A1), the concentration–response data was fitted to a 4-parameter logistic fit using Prism v5.04 to yield its IC_50_ in each assay. As sc1o was weakly active, the inhibition percentage at 100 µM was calculated and reported. (**b**) Inhibition of *h*ERG by sc1o was calculated using the Predictor *h*ERG fluorescence polarization assay. As a positive control E-4031, a blocker of *h*ERG-type potassium channels, yielding 100% inhibition and as negative control 100 nl of 100% v/v DMSO yielding 0% inhibition were added.

**Figure 3:**
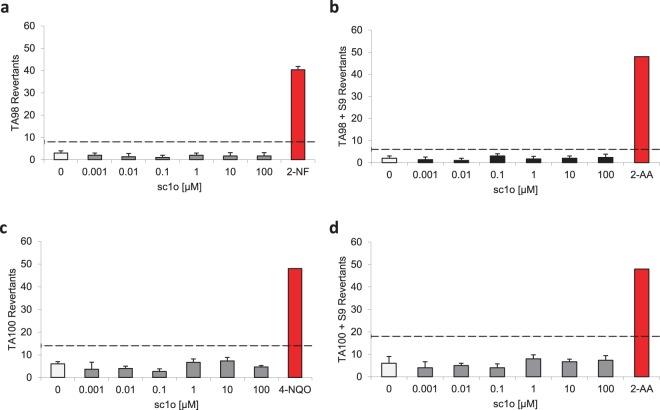
The AMES test reveal no mutagenic potential for sc1o. The AMES test was conducted with two *Salmonella typhimurium* strains TA98 (**a/b**) and TA100 (**c/d**) in the presence (**b/d**) or absence (**a/c**) of liver homogenate S9 to simulate the metabolic conversions of sc1o with liver enzymes. The AMES MPF 98/100 assay from Xenometrix was performed as described by the supplier. Data were analysed with the AMES MPF calculation sheet provided by Xenometrix. The experiment was performed once in triplicates as suggested by the supplier. The dashed line indicate 2 fold increase over baseline. Abb. BL, base line, 2-AA, 2-aminoanthracene; 2-NF, 2-nitrofluorene; 4-NQO: 4-nitroquinoline-N-oxide.

To further characterize its safety, possible off-target effects of sc1o were determined. These studies made use of HDAC1, HDAC3 and HDAC6 assays. Notably, sc1o did not significantly inhibit these HDACs (Fig. 2a). Furthermore, potential effects on PDE4, PDE7 and PDE8 were analysed. Sc1o also did not interact with these PDEs (Fig. 2a). Moreover, we analysed whether sc1o modulates CYP enzymes (CYP3A4, CYP1A2, CYP2D6). Sc1o did not significantly inhibit CYP enzymes, nor did it mediate CYP induction (Fig. 2a). A moderate inhibition of *h*ERG function was observed (40 ± 9% inhibition at 3 µM) which is within acceptable limits^15^ (Fig. 2a/b). We investigated also whether sc1o has mutagenic potential. For this purpose, we tested sc1o in the *Salmonella typhimurium* strains T100 and T98. To simulate metabolic conversion of sc1o by liver enzymes, the compound was incubated with T100 and T98 in the presence of the liver homogenate S9. Sc1o was negative in all AMES mutagenic assays (Fig. 3). These data indicate that sc1o has a good *in-vitro* safety and off-target profile.

### No disturbance of cell barrier integrity by sc1o

**Figure 4:**
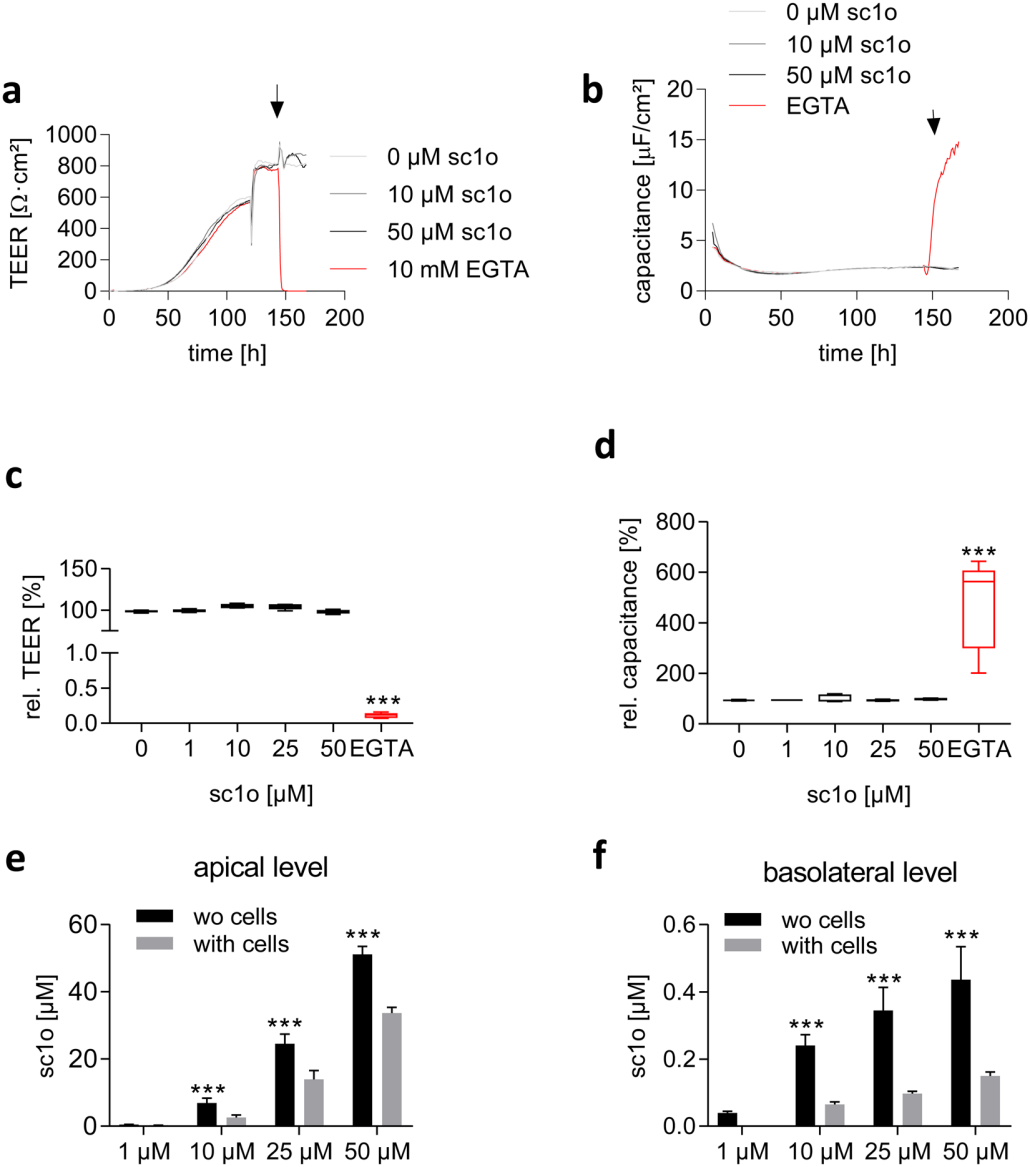
Influence of sc1o on Caco-2 cell barrier integrity and the pass-through rate of sc1o. (**a/b**) TEER (**a**) and capacitance (**b**) as a function of time in the presence or absence of different sc1o concentrations (0, 10, 50 µM) or of 10 mM EGTA. The arrow indicates the addition of buffer containing sc1o or EGTA. The data is a representative example from one of three independent experiments performed in duplicate. Caco-2 cells were cultured for 6 days on permeable transwell filters. At day 6, medium with sc1o at various concentrations or EGTA was added. The TEER and capacitance values were followed in a cellZscope2 device. (**c/d**) To calculate the effect of sc1o on the TEER or capacitance value, the values shortly before adding sc1o was set to 100%. The TEER or capacitance values 20 h after adding the sc1o were used to calculate the sc1o effects. (**e/f**). To determine the pass-through rate of sc1o, Caco-2 cells were cultured for 6 days on permeable transwell filters. On day 6, medium with sc1o at various concentrations was added, and after 24 h, the apical and basolateral medium were collected. As control transwell filters without cell barriers were incubated for 24 h with the indicated sc1o concentrations. The sc1o concentrations in the apical and basolateral medium was determined using LC-MS/MS. For statistical analysis, two-way ANOVA with Sidak’s multiple (a,c,e,f) comparison or one-way analysis and Dunnett’s multiple comparisons test (b,d) was used. *p < 0.05, ***p < 0.001 indicate significant differences between sc1o/EGTA and control samples or samples with and without cell barrier.

In multicellular organisms, the epithelial cell layers serve as functional barriers. Key components of epithelial cell barriers are the junctions between adjacent cells. Tight junctions are particularly relevant for the active barrier function of the cell layer, since they regulate the passage of molecules across the barrier by selectively opening and closing in response to various signals from inside and outside the cells. Drugs have to pass through these tissue barriers. A direct correlation exists between the permeability of a cell layer and its electrical resistance, i.e. transepithelial electric resistance (TEER). Therefore, the TEER value can be used to quantify the tightness of the barrier. Additionally, electrical capacitance can be detected, which provides information on the morphology of the membrane, such as the expression of microvilli and other membrane extrusions. Caco-2 cell barriers were generated on porous membranes, and TEER and capacitance were determined to characterize whether sc1o influences the integrity of the cell barrier. Ethylene glycol-bis(β-aminoethyl ether)-N,N,N′,N′-tetraacetic acid (EGTA) was used as a positive control since EGTA leads to a depletion of extracellular Ca^2+^, which in turn causes a disassembly of tight junctions^16^. As expected, adding EGTA led to a drop in TEER readings and to an increase in the capacitance (Fig. 4a/b). To quantify the alteration of TEER and capacitance, the TEER/capacitance values 20 h after adding the various stimuli (EGTA, sc1o) were compared with the TEER/capacitance value before adding the stimuli. EGTA reduced the relative TEER to 0.12 ± 0.03% and increased the relative capacitance to 481 ± 168% (Fig. 4c/d). Sc1o altered neither the TEER nor the capacitance value (Fig. 4a-d).

### Low transport rate of sc1o in the Caco-2 cell barrier assay

To determine the permeability of cells to sc1o, the amount of sc1o that passes through a Caco-2 cell barrier was determined. For this, a Caco-2 cell barrier was generated on a porous membrane and covered with various concentrations of sc1o for 24 h. As a control, porous membranes without a cell barrier were incubated with sc1o for 24 h. The concentrations of sc1o in the basolateral and apical media were determined using LC-MS/MS. In the apical compartment, sc1o concentrations were higher in wells without than in wells with a cell barrier. These data indicate that sc1o was bound to the membrane and/or penetrated into the cells (Fig. 4e). Furthermore, in the basolateral compartment, sc1o concentrations were higher in wells without a cell barrier than in wells with one, indicating that the cell barrier was functional (Fig. 4f). In the basolateral compartment, a concentration-dependent increase in sc1o was detected, indicating that sc1o is able to penetrate through the cell barrier (Fig. 4f). However, only one hundredth of the administered 10 µM sc1o was found in the basolateral compartment. Bearing in mind that, in the apical compartment, sc1o concentration was low in the presence of cells, these findings indicate that the cells absorbed or accumulated sc1o in some way. Further studies are necessary to find out whether sc1o is bound to the membrane or is taken up by the cell where it can exert activity.

## Discussion

Sc1o was recently identified as a new and promising candidate for the treatment of malaria^9^. The next step on the path towards drug development includes generating an *in-vitro* safety profile and a prediction of potential bioavailability. Our results reveal that sc1o did not disturb Caco-2 cell barrier integrity but it is characterized by a low permeability potential. Furthermore, sc1o has no off-target effects, no mutagenic potential (AMES test) and no apoptosis induction potential. However, at >10 µM, sc1o reduced cell viability of HCT 116 cells, possibly by inducing a G1 block, an action linked with reduced cell proliferation in a cancer cell line.

Hughes *et al*. also stated that cytotoxicity can be considered tolerable if the cytotoxic concentration is at least 50-fold higher than the effective concentration^10^. We observed cytotoxic effects at 10 µM in a cancer cell line, whereas sc1o had an IC_50_ of 4.1 ± 1.6 nM against the malaria parasite *P. falciparum* (strain 3D7)^9^. Sc1o therefore, meets the generally accepted criteria for a lead compound, as reported in the literature^10^, because sc1o up to 100 µM was largely inactive (yielding <<50% inhibition) in all but one of the safety and off-target assays. The only suggestion of a liability detected for sc1o was associated with *h*ERG (40 ± 9% inhibition at 3 µM compound concentration) which is not sufficient to prevent further development of the compound. The G1 block detected in HCT-116 cells was obtained at 10 µM sc1o which is also a concentration 2000 times higher than the IC_50_ (around 5 nM) for the eradication of *P. falciparium*.

Caco-2 permeability of drugs is a frequently used system to estimate transport across the intestinal epithelium, which is important for drug absorption from the gut. Drugs can use the paracellular and/or the transcellular route to penetrate through the colon wall. For the paracellular route the drug have to migrate through the apical and basolateral membrane and for the transcellular route drugs have to traverse the tight junctions. The Caco-2 cell barrier assays performed in this study reflect both the para- and the transcellular routes. The effect of sc1o on the transcellular route was investigated using the TEER measurements, whereas the trans- and paracellular routes were investigated with the transport assay. Sc1o did not alter TEER values, indicating that sc1o cannot use the transcellular route to penetrate trough the colon wall. Sc1o showed also a low transport rate, indicating that it also did not use the transcellular route adequately. The low permeability and possibly high protein binding is in line with a low serum concentration of about 2 µM after intraperitoneal administration of 100 mg/kg sc1o in mice^9^. To improve the bioavailability of sc1o, two options are available. Synthetic modification of the molecular structure and/or suitable formulation, as the absorption of many drug molecules with poor permeability can be improved with excipients^17^. For sc1o, it has already been shown that the steroid and the hydroxyarylmethylamino moieties are essential for antimalarial activity, supporting a chelate-based quinone methide mechanism involving metal or haem bioactivation^9^. Alteration of the structure is thus, likely to reduce the antimicrobial efficacy. To increase the bioavailability of sc1o without reducing its antimicrobial efficacy, a suitable formulation such as solid dispersions, offers many benefits over conventional drug delivery approaches^18^.

Taken together, our findings indicate that sc1o has a good *in-vitro* safety profile and a suitable formulation should ameliorate its low permeability.

## Materials and methods

### Cells and reagents

HCT 116 cells were cultured in McCoy’s 5 A (modified) medium supplemented with 10% FCS. RAW264.7 macrophages were cultured in RPMI1640 GlutaMAX medium supplemented with 10% FCS. HEK293 cells were cultured in DMEM supplemented with GlutaMAX, 10% heat-inactivated FCS. Caco-2 cells were from Sigma and were cultured in EMEM medium supplemented with 10% FCS, L-glutamine, and non-essential amino acids (Sigma Aldrich, M7145). All media contain 1% penicillin/streptomycin, and the cells were cultured at 37 °C in a 5% CO_2_ atmosphere. Sc1o was dissolved in DMSO and further diluted in media (c_stock_ = 25 mM, maximal DMSO concentration during experiments 0.3% v/v). EDTA was from Sigma Aldrich (Schnellendorf, Germany). Lucifer Yellow (sc-215269) was from Santa Cruz. Standard inhibitors included trichostatin A (Sigma-Aldrich, USA), E-4031 (BML-KC158–0005, Enzo Life Sciences, Inc. NY, USA), valinomycin (CAYM10009152–25, Cayman Chemical, Ann Arbor, MI, USA), alpha-naphthoflavone (N5757–1G, Sigma-Aldrich, USA), quinidine (Q3625–5G, Sigma-Aldrich, USA), ketoconazole (K1003, Sigma-Aldrich, USA), and 3-isobutyl-1-methylxanthine (IBMX) (I7018, Sigma-Aldrich, USA). Human recombinant enzymes included C-ter-His-FLAG-HDAC1 (50051), HDAC-3/NcoR2 (50003) and N-ter-GST-HDAC-6 (50006) from BPS Bioscience (San Diego, CA, USA). PDE4A1 (AMS.60021), and PDE7A1 (AMS.60070) and PDE8A1 (AMS.60080) were purchased from AMSBIO. Assay kits used included Predictor *h*ERG (Thermo, Waltham, MA, USA), P450-Glo (Promega Corp., Madison, WI, USA), and HDAC-Glo Class I/II kits (Promega Corp., Madison, WI, USA).

### Cell viability/proliferation assays

For the WST-1 assays, 2 × 10^4^ HCT 116 cells were incubated for 24 h at 37 °C. Sc1o (0–50 µM) and control (DMSO) were added to the cells and mixed. After a 24 h incubation step, 10 µl WST-1 reagent (Sigma Aldrich, Germany) was added, mixed, and incubated at 37 °C for 60 min. Absorbance was measured at 450 nm and at 650 nm (reference) using an EnSpire plate reader (PerkinElmer, Waltham, MA, USA). The absorbance at 450 nm was normalized with the absorbance at 650 nm. The sample values were corrected with the background wells (wells with medium and without cells). To calculate cell viability, the absorbance of DMSO-treated cells was set to 100%, and the sc1o samples were correlated to the absorbance of DMSO value.

### Apoptosis assay

For this assay, 2 × 10^4^ HCT 116 cells were seeded in a black poly-D-lysine-coated 96-well plate and were incubated for 24 h at 37 °C. The culture medium was replaced with 100 µl DMEM medium without phenol red and was supplemented with 10% FCS and 1% penicillin/streptomycin. Sc1o (0–50 µM) or control (DMSO) were added to the cells and incubated for 24 h at 37 °C. One µl of CellEvent Caspase-3/7 green detection reagent (1:10 diluted in DMEM medium without phenol red) was added and incubated for 90 min at 37 °C (without CO_2_). Afterwards, 1 µl of DRAQ5 (1:25 diluted in DMEM-medium without phenol red) was added, and the cells were incubated for 30 min at room temperature (RT). An image was taken using the ImageXpress micro-confocal high-content imaging system (Molecular Device, San Jose, USA). Cell nuclei were stained in red (Cy5 channel); apoptotic cells appeared in green (FITC channel). The percentage of dead cells was determined using the ‘live/dead’ analysis tool from Molecular Device by calculating the ratio of apoptotic cells (green) to all cells (red). Four different measuring sites in the well were analysed, and the average value was used for data presentation.

### Cell cycle assay

For this assay, 2 × 10^4^ HCT 116 were seeded in a 96-well cell culture plate and incubated for 24 h at 37 °C. The culture medium was replaced with 100 µl medium containing sc1o in various concentrations or control substances. As a negative control, medium with 10% FCS was used. G1/S-block was induced via medium without 10% FCS. Twenty µM curcumin and 0.2 µM staurosporine were used to induce G2- and S-block, respectively. After an incubation step of 24 h at 37 °C, cells were harvested, suspended in 200 µl sample buffer (1 g glucose/1 l phosphate-buffered saline (PBS) without calcium or magnesium), mixed, centrifuged (200 g, 4 min, 4 °C), and the supernatant was discarded. This step was repeated once. Cells were fixed with 150 µl of ice-cold 70% ethanol overnight (>18 h) at 4 °C. Cell pellet was washed with sample buffer, resuspended in 100 µl staining buffer (20 µ/ml propidium iodide and 0.2 mg/ml RNase in sample buffer) and incubated for 40 min at RT. Samples were measured within 24 h in a MACSQuant analyser (Miltenyi Biotec GmbH, Bergisch Gladbach, Germany). Cell cycle distribution was determined using FlowJo software.

### Mitochondrial toxicity assay

This assay made use of the MitoTracker Red CMXRos dye (Thermo, Waltham, MA, USA), which stains mitochondria in live cells, and its accumulation is dependent upon membrane potential. The renal carcinoma 786–0 cell line was harvested from a 75 cm^2^ flask at 80% confluency by washing it once using 5 ml RT PBS and incubating with 1 ml trypsin 0.05% / EDTA 0.02% for 3 min. Cells were suspended in 10 ml pre-warmed cell culture media (RPMI-1640 supplemented with 10% FCS, 100 U/ml penicillin and 100 µg/ml streptomycin) and counted using a Scepter (Merck Millipore, Germany). Cells were diluted to 7.5 × 10^4^ cells/ml, and 20 µl of this suspension was added to each well of a 384-well plate. Cells were incubated for 36 h at 37 °C and 5% CO_2_. Sc1o was added using a pre-dilution plate. The positive control was valinomycin at a final concentration of 1 µM, with the negative control (DMSO) at the same concentration (v/v). After incubation, 10 µl of a 200 nM solution of MitoTracker Red CMXRos in pre-warmed cell culture media was added to each well, and the cells were incubated for an additional 45 min at 37 °C and 5% CO_2_. MitoTracker Red CMXRos uptake was measured using an Opera imaging system. To facilitate automatic image analysis, the layout containing the compound area, as well as the valinomycin and DMSO control areas, was created and stored. A sub-layout of five evenly dispersed fields per well was used. These settings also included a measurement height of 1 µm, which was stored in an exposure file format. By using the stored settings and files, an automated run was repeatedly created and executed. The images obtained were transferred to the file server and uploaded into Columbus 2.4.0 using the built-in helper function and were analysed therein.

### HDAC assays

Inhibition of histone deacetylase (HDAC1, HDAC3 and HDAC6) enzymes was measured using the homogeneous, single addition, bioluminogenic HDAC-Glo I/II assay (Promega Corp., USA). Briefly, sc1o in 11-point concentration-response format in triplicate (100 nl of 1 mM solution in 100% v/v DMSO), positive controls (trichostatin A, final concentration of 1 μM and 1% v/v DMSO) yielding 100% inhibition, and negative controls (100 nl of 100% v/v DMSO) yielding 0% inhibition were added to each well of a 384-well microtitre plate by using the Echo 550 liquid handler. Assays were initiated by adding 10 µl/well of the HDAC-Glo I/II assay reagent (prepared by rehydrating lyophilized HDAC-Glo I/II substrate in 10 ml HDAC-Glo I/II assay buffer and 10 μl developer reagent) and were mixed briefly via orbital shaking (500–700 rpm). The luminescence was measured at steady-state signal:background, which was achieved after 20 min incubation at RT using an EnVision Multilabel 2103 reader (PerkinElmer, Waltham, MA, USA).

### PDE assays

Inhibition of phosphodiesterase (PDE4A1, PDE7A1 and PDE8A1) enzymes was measured using the LANCE Ultra cAMP assay (PerkinElmer, Waltham, MA, USA). Briefly, sc1o in 11-point concentration-response format in triplicate (100 nl of 1 mM solution in 100% v/v DMSO), positive controls (IBMX with final concentration of 100 µM and 1% v/v DMSO) yielding 100% inhibition and negative controls (100 nl of 100% v/v DMSO) yielding 0% inhibition were added to each well of a 384-well microtitre plate by using the Echo 550 liquid handler. Ten nM of cAMP solution in assay buffer (5 µl/well) was subsequently added and incubated for 45 min at RT. This was followed by Eu-cAMP tracer in detection buffer (to stop the enzymatic reaction), including 2 mM of IBMX (5 µl/well) and ULight-anti-cAMP in detection buffer (5 µl/well), and was incubated for 1 h at RT, after which the TR-FRET signal was measured using an EnVision Multilabel 2103 reader (PerkinElmer, Waltham, MA, USA).

### CYP assays

The inhibition of CYP (3A4, 1A2 and 2D6) and induction of CYP were measured using the luminescence-based P450-Glo (Promega Corp., USA) assay system. Briefly, sc1o in 11-point concentration-response format in triplicate (100 nl of 1 mM solution in 100% v/v DMSO), positive controls (3A4, ketoconazole; 1A2, α-naphthoflavone; 2D6, quinidine with a final concentration of 1 µM and 1% v/v DMSO) yielding 100% inhibition, and negative controls (100 nl of 100% v/v DMSO) yielding 0% inhibition were added to each well of a 384-well microtitre plate by using the Echo 550 liquid handler. This was followed by adding the CYP/substrates (5 µl/well) and was incubated for 30 min at 37 °C. Reactions were initiated by adding an NADPH regeneration system (5 μl/well). The reactions were stopped by adding luciferin detection reagent (10 μl/well), followed by an additional 30 min incubation at 37 °C with the luminescence signal detected using an Infinite M1000 PRO plate reader (Tecan, Männedorf, Switzerland).

### *h*ERG assay

Inhibition of *h*ERG was measured using the Predictor *h*ERG fluorescence polarisation assay (Thermo Fisher Scientific, Waltham, MA, USA). Briefly, sc1o in 11-point concentration-response format in triplicate (100 nl of 1 mM solution in 100% v/v DMSO), positive controls (E-4031, a blocker of *h*ERG-type potassium channels) yielding 100% inhibition, and negative controls (100 nl of 100% v/v DMSO) yielding 0% inhibition were added to each well of a 384-well microtitre plate by using the Echo 550 liquid handler. This was followed by adding homogenised membrane solution (5 µl/well) and a 1 nM final concentration in assay (5 µl/well). The plates were incubated for 2 h at RT in a humidity-controlled incubator, and fluorescence polarisation was measured using an EnVision Multilabel 2103 reader (PerkinElmer, Waltham, MA, USA).

### AMES test

The AMES test was conducted with two *Salmonella typhimurium* strains TA98 and TA100 in the presence or absence of liver homogenate S9 to simulate the metabolic conversions of sc1o with liver enzymes. The Ames MPF 98/100 from Xenometrix was used as described by the supplier. The principle of the test is based on mutated bacteria strains. Point mutations were made in the histidine operon of *Salmonella typhimurium*, rendering the bacteria incapable of producing the corresponding amino acid. These mutations resulted in histidine-deficient organisms that cannot grow unless histidine is supplied. When a mutagenic event occurs, base substitutions or frameshifts within the gene may cause a reversion to amino acid prototrophy. These reverted bacteria will then grow in histidine-deficient media. After exposure with increasing concentrations of sc1o or with positive controls (2 μg/ml for 2-NF (TA98), 0.1 μg/ml for 4-NQO (TA100), 2.5 μg/ml (TA100), and 1.0 µg/ml (TA98) for 2-AA red, the cultures were diluted in pH indicator medium lacking histidine and aliquoted into a 384-well plate. Within two days, cells that had undergone reversion to amino acid prototrophy grew into colonies. Bacterial metabolism reduces the pH of the medium, changing the color of that well. Finally, the number of wells containing revertant colonies were counted for each dose and compared to a solvent (negative) control. The experiment was conducted once in triplicate. The data was analysed with the AMES MPF calculation sheet provided by Xenometrix. Fold induction over the baseline was the ratio of the mean number of positive wells for the dose concentration divided by the baseline. The baseline is obtained by adding one standard deviation to the mean number of positive wells of the solvent control. Compounds with mutagenic potential are characterized by revertant numbers above the baseline

### Cell barrier model

Twenty thousand CaCo-2 cells were seeded on 24-well ThinCerts (pre-coated with FCS for 30 min). In the lower compartment (basolateral), 1 ml culture medium per well was added. As a control, half of the ThinCerts were not seeded with cells. The ThinCerts were transferred to the cellZscope2 (nanoAnalytics). Two hundred µl of medium was added to the ThinCerts to reach a final volume of 300 µl in the upper compartment (apical). The TEER and capacitance were measured every hour. After 5 days, the TEER reached a constant value of 700–900 Ohm*cm² and the medium was replaced with EMEM with 2% FCS, 2 mM L-glutamine, 1x non-essential amino acids, and 110 nM hydrocortisone. The TEER and capacitance were measured every hour for 1 day. Sc1o (0 µM, 1 µM, 10 µM, 25 µM, 50 µM) diluted in EMEM with 2% FCS, 2 mM L-glutamine, 1x non-essential amino acids, and 110 nM hydrocortisone were added to the apical compartment. As a control, 10 mM of EGTA was used. The cellZscope2 module was transferred into the incubator for 24 h, and every 30 min the TEER and capacitance were detected. TEER and capacitance values at the time point of adding the compound solution were used as references to calculate the alteration (t0h-value). For the determination of the transport rate the Caco-2 cell barrier was generated as mentioned above. Sc1o (0 µM, 1 µM, 10 µM, 25 µM, 50 µM) diluted in EMEM with 2% FCS, 2 mM L-glutamine, 1x non-essential amino acids, and 110 nM hydrocortisone were added to the apical compartment and shaked at 40 rpm. At time point 0h and after 24 h the medium of the basolateral and apical compartment was collected and stored at −20 °C and analysed by LC-MS/MS.

### Determination of sc1o via LC-MS/MS

For the quantification of sc1o, 20 µl samples were mixed with 20 µl acetonitrile, 20 µl of the internal standard (sc1c, 200 ng/ml in acetonitrile) and 150 µl methanol. The mixture was vortexed for 1 min and centrifuged at 20,000 g for 3 min, and the clear supernatant was transferred to an autosampler vial. Samples in which a high concentration of sc1o was expected were diluted by a factor of 50 with 25% DMSO in water, and 20 µl of the diluted sample was processed as stated. For calibration and quality control samples, 20 µl water were mixed with 20 µl standard or quality control working solution and processed as stated. The amount of sc1o was analysed via liquid chromatography coupled to tandem mass spectrometry. An Agilent 1260 series binary pump (Agilent technologies, Waldbronn, Germany) equipped with a Zorbax Eclipse Plus C18 UHPLC column (50 mm × 2.1 mm ID, 1.8 μm, Agilent technologies, Waldbronn, Germany) was used for chromatographic separation. Mobile phase A was water with 0.2% formic acid and 10 mM ammonium formate, whereas mobile phase B was acetonitrile/isopropanol/acetone (50:30:20, v/v/v) with 0.2% formic acid. The gradient program started with 65% mobile phase A for 0.5 min, then A was decreased within 1.5 min to 0%, held at 0% for 1 min and increased to 65% again within 0.1 min. Total run time was 5 min. The MS/MS analyses were performed using a triple quadrupole mass spectrometer QTrap 5500 (Sciex, Darmstadt, Germany) equipped with a Turbo V ion source operating in positive electrospray ionization mode. The analysis was done in multiple reaction monitoring (MRM) mode. Information on the recorded mass transitions for the analyte and internal standard is given in Supplemental Fig. 1. Data was acquired using Analyst Software V 1.6.2 and quantified with MultiQuant Software V 3.0.2 (both Sciex, Darmstadt, Germany), employing the internal standard method (isotope dilution mass spectrometry). The calibration curve was calculated via linear regression with 1/x weighting. Variations in the accuracy of the calibration standards were less than 15% over the whole range of calibration, except for the lower limit of quantification where a variation in accuracy of 20% was accepted.

### Statistics

Results are presented as means ± standard errors (SEM). The data was analysed with one-way or two-way analysis of variance (ANOVA) and with Dunnet’s or the Shapiro-Wilk comparison test. For all calculations and creation of graphs, GraphPad Prism 8 was used and p < 0.05 was considered the threshold for significance. For the safety profile and off-target studies of sc1o (mitochondrial toxicity, CYP 3A4, CYP 1A2, CYP 2D6 inhibition, CYP induction, *h*ERG, HDAC1, HDAC3, HDAC6, PDE4A1, PDE7A1, and PDE8A1), the concentration–response data was fitted to a 4-parameter logistic fit using Prism v5.04 to yield its IC_50_ in each assay. As sc1o was weakly active, the inhibition percentage at 100 µM was calculated and reported.

## Supplementary information

Supplementary information

## Data Availability

The data from any performed experiment is available from the corresponding author.
