# Supplementary material for: In-vitro safety and off-target profile of the anti-parasitic arylmethylaminosteroid 1o
Source: Sci Rep. 2020 May 5;10:7534. doi: 10.1038/s41598-020-64382-w (PMC7200784; doi:10.1038/s41598-020-64382-w)
Supplement: Supplementary file 1 — Supplementary information [file 41598_2020_64382_MOESM1_ESM.pdf]

Supplements

In-vitro safety and off-target profile of the anti-parasitic arylmethylaminosteroid 1o

Leonard Blum[1,2], Sheraz Gul[4], Thomas Ulshöfer[1], Marina Henke[1], Reimar Krieg[5], Isabell Berneburg[3], Dominique Thomas[2], Sandra Trautmann[2], Jennifer Kurz[1], Joachim Geyer[6], Gerd Geisslinger[1,2], Katja Becker[3], Michael J. Parnham[1] and Susanne Schiffmann[1*]

[1] Fraunhofer Institute for Molecular Biology and Applied Ecology IME, Branch for Translational Medicine and Pharmacology (TMP), Theodor-Stern-Kai 7, 60596 Frankfurt am Main, Germany.

[2] *pharmazentrum frankfurt*/ZAFES, Department of Clinical Pharmacology, Goethe-University Hospital Frankfurt, Theodor-Stern-Kai 7, 60590 Frankfurt/Main, Germany.

[3] Biochemistry and Molecular Biology, Interdisciplinary Research Center, Justus-Liebig-University, Heinrich-Buff-Ring 26-32, 35392 Giessen, Germany.

[4] Fraunhofer Institute for Molecular Biology and Applied Ecology IME – ScreeningPort, Schnackenburgallee 114, 22525 Hamburg, Germany.

[5] Department of Anatomy II, University Hospital Jena, Teichgraben 7, 07743 Jena, Germany

[6] Faculty of Veterinary Medicine, Institute of Pharmacology and Toxicology, Justus-Liebig-University Schubertstraße 81, 35392 Giessen, Germany.

Compound	Q1 [m/z]	Q3 [m/z]	DP [V]	EP [V]	CE [V]	CXP [V]	Comment
1o	442.27	128.10	291	10	101	6	Quantifier
1o	442.27	157.10	291	10	53	8	Qualifier
1o	442.27	173.20	291	10	49	4	Qualifier
1c	392.25	121.00	71	10	25	2	Quantifier
1c	392.25	65.10	71	10	93	16	Qualifier

Supplemental Figure 1: Mass transitions of analyte and the internal standard.
